# System Derived Spatial-Temporal CNN for High-Density fNIRS BCI

**DOI:** 10.1109/OJEMB.2023.3248492

**Published:** 2023-03-16

**Authors:** Robin Dale, Thomas D. O'sullivan, Scott Howard, Felipe Orihuela-Espina, Hamid Dehghani

**Affiliations:** ^1^ University of Birmingham 1724 B152TT Birmingham U.K.; ^2^ University of Notre Dame 6111 Notre Dame IN 46556 USA

**Keywords:** fNIRS, brain-computer interface, neural network, machine learning, CNN

## Abstract

An intuitive and generalisable approach to spatial-temporal feature extraction for high-density (HD) functional Near-Infrared Spectroscopy (fNIRS) brain-computer interface (BCI) is proposed, demonstrated here using Frequency-Domain (FD) fNIRS for motor-task classification. Enabled by the HD probe design, layered topographical maps of Oxy/deOxy Haemoglobin changes are used to train a 3D convolutional neural network (CNN), enabling simultaneous extraction of spatial and temporal features. The proposed spatial-temporal CNN is shown to effectively exploit the spatial relationships in HD fNIRS measurements to improve the classification of the functional haemodynamic response, achieving an average F1 score of 0.69 across seven subjects in a mixed subjects training scheme, and improving subject-independent classification as compared to a standard temporal CNN.

## I. Introduction

Functional near-infrared spectroscopy (fNIRS) is a neuroimaging technique which affords safe, cheap and non-invasive measurement of human brain activity, while allowing a higher degree of mobility to the user as compared to other functional imaging technologies such as fMRI. fNIRS hardware and processing techniques have advanced considerably over the past two decades of research, however the essential technique is unchanged: Near-Infrared (NIR) light (650–900 nm) emitted by LEDs or laser diodes attached to the scalp diffuses through the outer layers of the head (scalp, skull, cerebro-spinal fluid, and outer-cerebral grey and white matter), where it is absorbed by various chromophores including oxy- and deoxy- haemoglobin (HbO & Hb), and scattered by cellular and sub-cellular structures. A small proportion of the back-scattered photons are detected by photodetectors (optodes) on the scalp at distances of 5–50 mm from the source. The attenuation of the detected light over time is used to infer changes in Hb & HbO concentrations in the outer layer of the cortex, caused by the increased oxygen metabolism of firing neurons. This technique has been effectively applied to a wide variety of clinical and research problems, including cognitive neuroscience [1], detection and monitoring of psychiatric conditions whose symptoms include altered cognitive function, such as Alzheimer's [2] and schizophrenia [3], and as a method of brain-computer interface (BCI), in which a channel of communication is established between a user's measured brain signals and a computer [4].

Active BCI involves detecting and classifying deliberately generated brain activity in a specific region, most commonly the motor and prefrontal cortices [4]. Such systems afford the possibility to restore physical capabilities to people with neuromuscular impairments, such as amputees or sufferers of locked-in syndrome, by connecting the BCI to the controller of a robotic prosthesis [5]. However, due to the multiple-second delay in the haemodynamic response to neural activity (compared to EEG temporal resolution on the order of milliseconds), fNIRS may be more suited to a decision-making BCI paradigm, in which the user focuses activity on one of several time windows, each associated with a different option from a selection displayed on a screen; Recent work has shown fNIRS to be highly flexible and reliable mode of BCI within this paradigm [6]. Additionally, passive BCI for the purpose of mental state inference has been demonstrated using fNIRS for several promising applications including mental workload [7] and drowsiness detection [8], emotional [9], and preference [10] inference. fNIRS and EEG can also be used in conjunction, as hybrid BCI, providing complementary non-invasive measures of neural activity [11]. In this work, detection and classification of a bi-lateral finger-opposition task was selected for the BCI paradigm, as this reliably produces identifiable motor cortex activity with the system and data collection paradigm described in V-C. However, the proposed classification model is transferable to other BCI tasks and brain regions, where high-density (HD) measurements are available.

Functional classification of fNIRS data (as in BCI or diagnosis paradigms) generally involves the extraction of temporal features relating to a haemodynamic response from the set of time-series corresponding to the set of source-detector pairs, and is complicated by the presence of noise, systemic signals (primarily cardiac and respiratory) and individual differences in physiology. Accurate functional classification can be achieved using neural networks, which are trained on labelled datasets to simulate the statistical mapping between the boundary measurements and the underlying mental state. This work investigates one method to improve the performance of such models: a combination of a specific fNIRS probe geometry with a neural network architecture which enforces parameter sharing between channels with similar source-detector separations (SDSs), and positionally invariant feature extraction. By specialising the network architecture to better reflect the underlying physics, while allowing the parameters to be learned from data, the aim is to achieve more efficient training and more reliable classification.

### A. fNIRS Hardware

Swift advancement is being made in fNIRS hardware, with state-of-the-art systems trending toward miniaturisation, mobility, and modularity. Several wearable fNIRS devices have recently been developed for commercial sale (e.g. [12]) - marketed as brain training or meditation tools, and part of a general trend in wearable technology for biometric monitoring and optimisation. fNIRS BCI may be integrated in near-future virtual reality headsets (as EEG already has been [13]), to enhance user experience through neurofeedback, or to gather real-time data on users' mental states to be used, for example, in targeted advertising. Consequently, increasingly large and user-diverse datasets may soon become available to organisations whose products are widely adopted for either clinical or commercial use, enhancing the potential of data-driven processing and classification methods.

There are three main types of fNIRS instrumentation: continuous-wave (CW), frequency-domain (FD), and time-domain (TD), requiring progressively more complex hardware and processing procedures, and providing progressively greater information content regarding tissue optical properties. Conventional CW-fNIRS measures the attenuation of light intensity only, while the time resolved measurements of FD and TD additionally indicate the time-of-flight of emitted photons - via the phase component of the complex FD signal, and the temporal point spread function in TD – which can potentially be used to recover absolute values of absorption and reduced scattering coefficients ($\mu _{a}$ and $\mu _{s\prime }$).

This work focuses on HD FD-fNIRS, which is more widely used than HD-TD, and fills an important niche in the evolving fNIRS landscape, providing the greater information content of the time-resolved measurement without the full complexity and weight of TD [14]. Furthermore, while FD generally requires more elaborate hardware than CW, recent advances in hardware have shown that high performance lightweight FD-fNIRS is possible. For example, Yazdi et al. have recently presented an FD system on a chip, with comparable optical property fidelity to a standard NIRS Diffuse Optical Spectroscopy (DOS) system [15]. The benefits of FD-DOS for both image reconstruction and functional classification are well documented. Scholl et al. performed PCA on FD-data collected from the visual cortices of subjects watching movie scenes, and found that the combined intensity and phase signal captured more orthogonal signal dimensions over training and hold-out data than either intensity or phase alone [16]. Thompson et al. found that the inclusion of the phase measurement along with intensity significantly improved the accuracy of a regression-based classifier in a two-class finger-opposition task, achieving higher accuracy for each of twelve subjects and significantly higher accuracy at the group level [17]. Doulgerakis et al. have demonstrated additional benefits of FD-DOS for tomographic image reconstruction of the head via simulation, showing that incorporating both HD-intensity and phase measurements can provide higher image resolution [18], and increased depth sensitivity [19]. Stillwell et al. have developed a handheld broadband FD-DOS device for breast imaging, with the capability to sweep through six wavelength and hundreds of modulation frequencies, and a maximum sample speed of 36,600 Hz for amplitude and phase data at a single wavelength and single modulation frequency [20]. This technology could be integrated into future wearable fNIRS devices, providing richer data streams with many more data channels than the conventional dual-wavelength intensity.

### B. Hd-Dos

The arrangement of sources and detectors is a crucial consideration in fNIRS as it determines the regions of sensitivity of the measurement channels. Channels with source-detector separation (SDS) $>20\,mm$ are more sensitive to cortical changes, as the detected photons have on average penetrated deeper into the head, while short SDS channels are sensitive primarily to superficial changes in the scalp, which are dominated by systemic activity. Where multiple channels are used, the probe arrangement affects both the extent of the field of view, and the distribution of measurement sensitivity within it. State-of-the-art fNIRS systems are increasingly tending towards wireless, wearable probes with multiple sets of multi-distance channels to improve differentiation between functional and systematic perturbations, however there is still considerable variety in the SD arrangements employed.

The standard high-density (HD-) array used in Diffuse optical tomography (DOT) employs a tightly packed arrangement of sources and detectors to create a grid of overlapping multi-distance measurement channels across the region of interest, and can be used to reconstruct 3D maps of cortical haemodynamic changes with spatial resolution comparable to the BOLD response measured using fMRI [21]. Accurate real-time DOT is prohibited by system noise and computational cost, and so direct classification, without the intermediate reconstruction stage, is needed for real-time applications such as BCI. However, HD-array may still improve functional classification both by improving localisation of haemodynamic changes and distinction between superficial and cortical changes. Furthermore, it could reduce the effect of small changes in probe position relative to the ROI, which can be caused by variations in probe placement between sessions (especially in the case of non-expert users), and by variation in cortical response between users. fNIRS applications with sparse arrays require precise probe placement to target the ROI, and while effective techniques for precise probe placement have been developed [22] these are often only viable in a clinical or laboratory context. In this research a data-driven feature extraction technique is proposed which exploits the specific geometry of the HD-DOT array to enhance the performance of a neural network model for classifying brain function, showing that choices in probe geometry affect model design in addition to ROI.

### C. Spatial-Temporal Feature Extraction

Temporal feature extraction from fNIRS timeseries has traditionally been achieved through manually selected features such as peak amplitude, mean value, variance, slope, or skewness, which can be used as input to train a machine learning model such as support vector machine (SVM), K-nearest neighbours (KNN), or linear discriminant analysis (LDA) classifier. Many recent fNIRS-BCI studies have found neural network-based models which learn features directly from fNIRS time series data to achieve superior results compared to standard ML techniques [23], [24], [25], [26], [27]. Convolutional neural networks (CNNs) and Long Short Term Memory networks (LSTMS) in particular have become prominent among the most effective methods of classifying fNIRS data. Both of these architectures employ parameter sharing mechanisms to efficiently extract recurrent features from temporal data, thereby improving training efficiency compared to a fully-connected architecture. While LSTM is generally preferred for time-series forecasting, [26] found a 1D temporal CNN to outperform uni- and bi-directional LSTM, in addition to SVM, KNN & LDA models for a two-class BCI gait prediction task.

Multi-channel fNIRS data also contains spatial information related to the relative positions on the sources and detectors, especially in the case of HD arrangements, where multiple channels may have overlapping regions of sensitivity. CNNs can be used to extract spatial features from multi-channel fNIRS measurements. This requires the data to be input as an image, or set of images (in computer vision, images are represented as [width x height x 3(RGB)] matrices), and the arrangement of the data over the spatial and channel dimensions determines the features that the convolutional filters extract. Several techniques for representing fNIRS data as images have been proposed, the most basic being a 2D Matrix with shape [n_channels x n_timesteps] to input the data from an entire task period in a single image. Depending on the channel numbering scheme employed, the first dimension of the flat fNIRS image can correlate with SDS, or with position on the head, and therefore its gradient can contain information pertaining to degree and location of functional activation, enabling 2D convolution. Janani et al. have achieved 72.35 $\pm$ 4.47% accuracy on a four-class motor-imagery BCI task using a spectrogram representation of fNIRS data, where each row corresponded to the strength of a particular temporal frequency over multiple time windows, and these were stacked together to produce a 2D image corresponding to a 20 s task period [24].

Other studies have demonstrated the utility of producing fNIRS images from measurements from a single time-step according to the positions of the channels in the probe. Yang et al. trained a CNN to detect mild cognitive impairment (the clinical precursor to Alzheimer's Disease) in subjects completing three different mental tasks and found that the use of temporal-spatial feature maps produced a more reliable performance than either spatial or temporal features individually [28]. Their CNN used a spatially resolved 2D input layer, where the temporal information was captured by selected features such as mean and slope measurements over specific time periods. Saadati et al. constructed spatial images of fNIRS signals at each time-step by matching the locations of measurement channels on the scalp to pixel positions and interpolating the values of the pixels between channels [7]. Using these 2D images as input into a CNN, they achieved an 8% increase in accuracy on an n-back (a classic experiment for measuring mental workload) classification task, compared to a DNN model with flattened input layer.

Sommer et al. arranged HD-fNIRS measurements in two areas of interest corresponding to the left and right primary motor cortices, and within these regions according to the SDS [29]. Rolling window samples of these images were used to train a CNN-LSTM model to predict finger-opposition laterality and frequency, achieving average accuracy of 0.81 over eleven subjects. They conducted a SHAP analysis of the trained CNN-LSTM model, showing that the highest node activation values in the network corresponded to the expected contralateral motor cortex activation, and tended to be in neighbouring channels, further supporting the choice of CNN for extracting spatial features.

The results of the studies described above show that robust classification performance can be achieved with CNN trained on fNIRS images, especially where the data is arranged to correspond with the physical measurement space.

In this work it is argued that the geometry of the standard HD-DOT array (shown in Fig. 6) is uniquely suited to convolutional feature extraction. It affords the possibility to produce fully spatially resolved, multi-depth topographical images of the fNIRS measurements, without a heuristic interpolation function. These images can be input directly to the first convolutional layer of the classifier network to perform spatial and temporal feature extraction simultaneously. In short, the HD-array allows the raw fNIRS measurements to be treated like video data.

**Figure 1.**
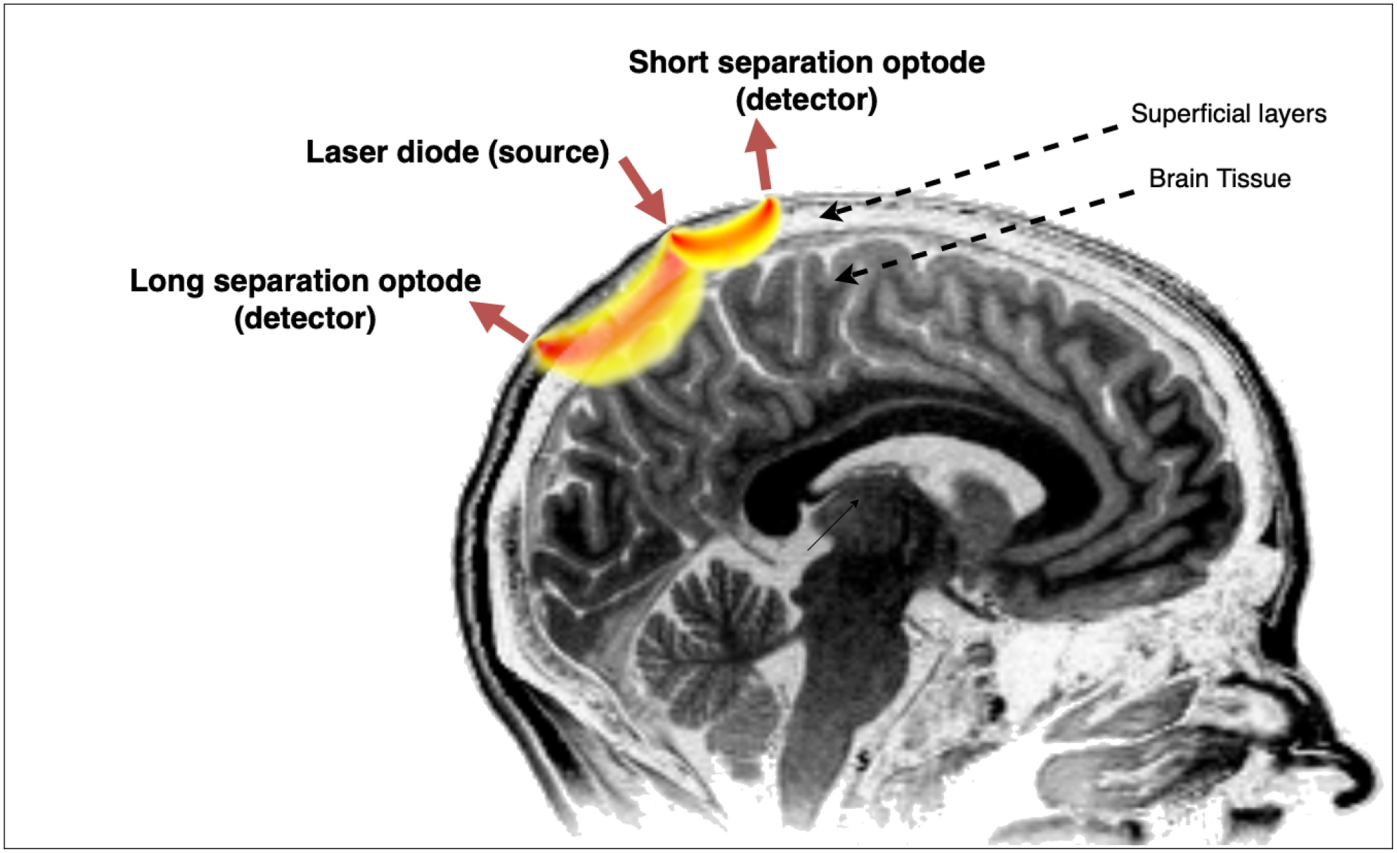
Illustration of the effect of source-detector separation (SDS) on the region of sensitivity of fNIRS channels.

**Figure 2.**
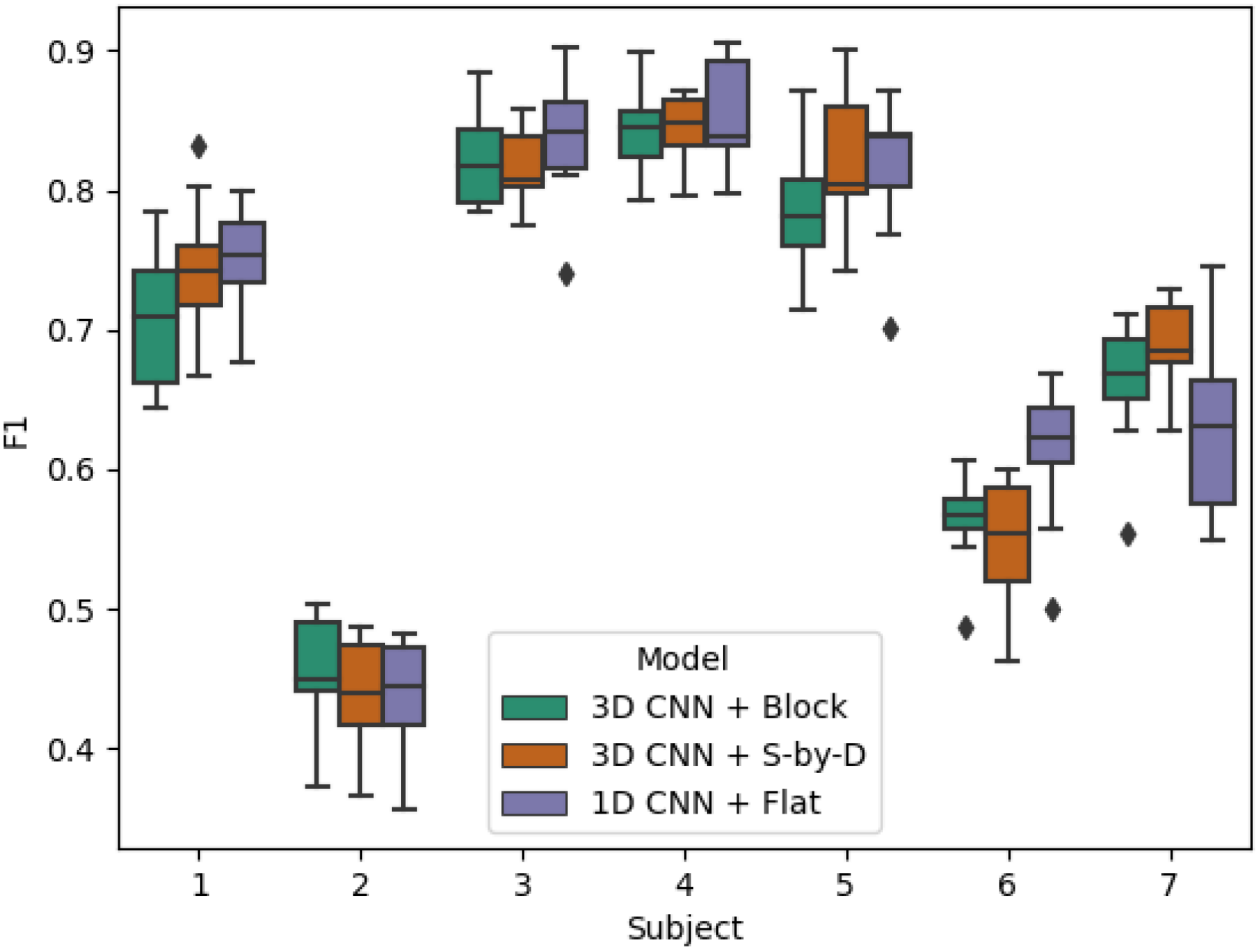
Classification results for each subject using the mixed subjects training scheme.

**Figure 3.**
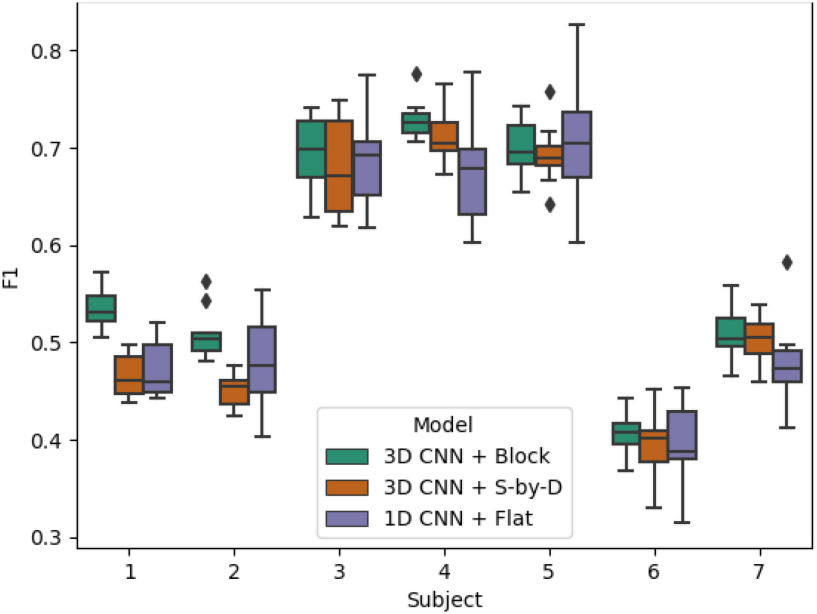
Classification results for each subject using the Subject-Independent training scheme.

**Figure 4.**
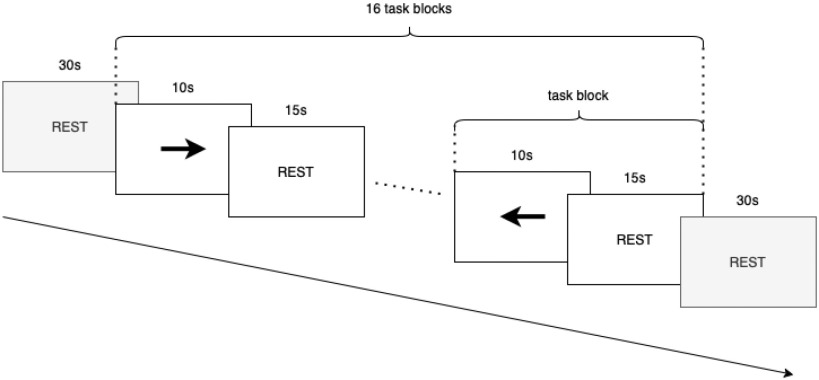
Schematic illustration of the finger tapping paradigm. Each experimental run consisted of an initial 30 s rest period, followed by 16 task blocks - each consisting of a 10 s task period followed by a 15 s rest period - followed by a final 30 s rest period.

**Figure 5.**
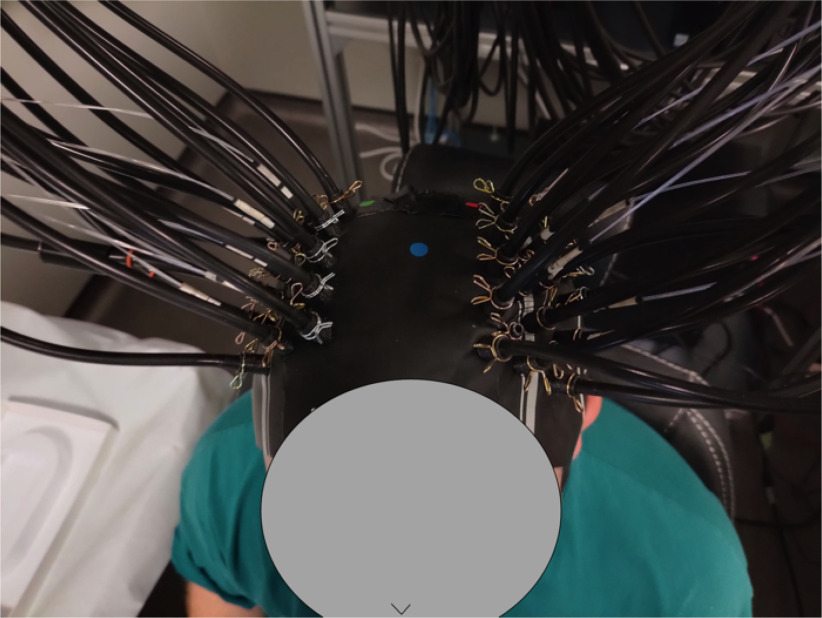
Subject wearing the high-density fNIRS cap with channels arranged over the motor cortex.

**Figure 6.**
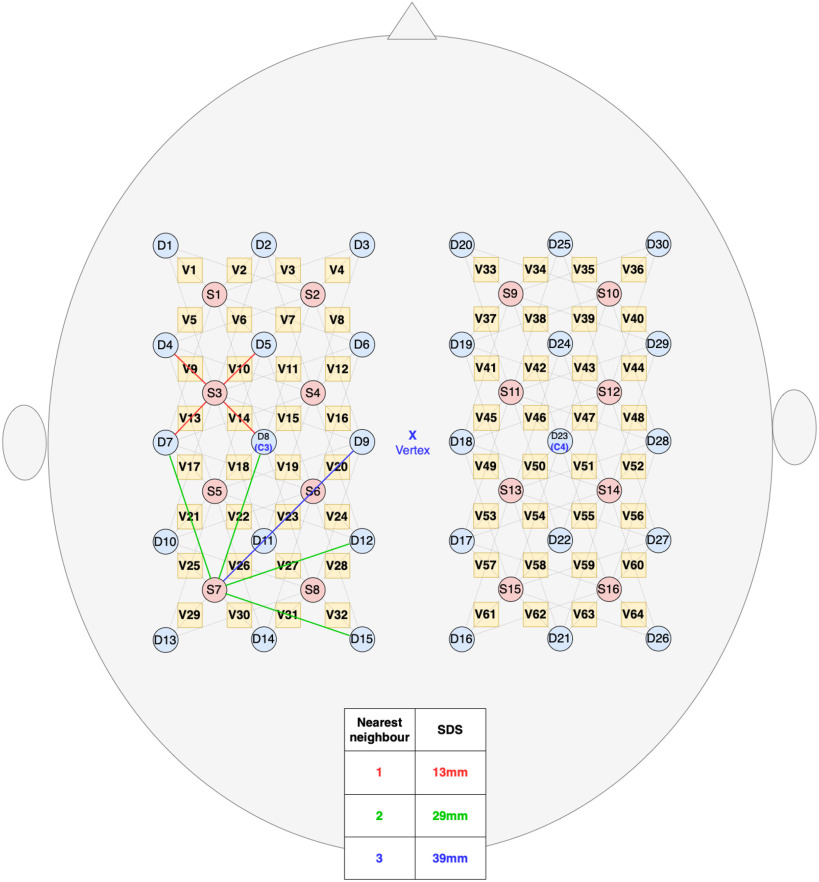
High-density source detector arrangement showing 1st, 2nd and 3 rd nearest neighbour distances and the voxels (yellow squares) where the channels' centre points overlap.

In this work, a model combining a 3D CNN architecture with a *Block* data structure for classifying fNIRS data is therefore proposed, based on a video classification model [30], with a 3D convolutional first layer to extract spatial and temporal features simultaneously. To quantify the effect of this data structure/architecture combination, the proposed model is compared to two other models: a 1D CNN with a 1D input (*Flat*) and fully connected feature extraction layer, and a 3D CNN whose input is a non-spatial representation of the same data (referred to as *Source-by-Detector*, or *S-by-D*).

The proposed model is demonstrated to accurately classify 10-second slices of processed fNIRS data based on task state (left hand activity/right hand activity/rest), necessary for a simple choice-making BCI paradigm, and could be adapted for other BCI tasks, given an appropriate training dataset.

## II. Results

Results are presented for three model + *data structure* combinations: *3D CNN + Block*, *3D CNN + S-by-D*, and *1D CNN + Flat* - for a three-class BCI motor-classification task described in Section V-B and for two training schemes: *mixed subjects* and *subject-independent*, described in Section?

### A. Mixed Subjects Results

In the mixed subjects scheme, the three models achieved similar F1 scores on each of the test subjects. There was high variability in F1 scores between the subjects (Fig. 2). For four out of the seven subjects, all the models achieved F1 Scores over 70%. The highest score for each model was approx 84% (subject 4), while scores associated with subject two were significantly lower than any other subject (approx 45%). A two-way ANOVA (analysis of variance) test revealed that there was not a statistically significant difference in average F1 score between any two models (F = 1.48, p = 0.23) (note: the F statistic here is the ratio of two variances revealed by the ANOVA test, and is different from the F1 score.

### B. Subject Independent Results

In the subject independent scheme, the F1 scores were generally lower than the mixed subjects scheme, with maximum accuracy around 70% compared to 80% (Fig. 3). Again, the models all achieved similar scores for each subject with considerable variation between subjects. *3D CNN + Block* improved classification compared to the other two models for all subjects except subject 5, with an average F1 score of 58.3%, 2.6% higher than *1D CNN + Flat* and 2.7% higher than *3D CNN + S-by-D*. A two-way ANOVA revealed that there was a statistically significant difference in sampled F1 scores between at least two models (F = 12, p = 1.3e-5). Post-hoc t-tests revealed a significant increase in sampled F1 scores for the 3D CNN+Block method compared to both the 1D CNN+Flat (F = 3.78, p = 2.3e-4), and 3D CNN+S-by-D (F = 5.19, p = 7.4e-7).

## III. Discussion

In this work, the use of spatially resolved HD-FD-fNIRS data to train a 3D CNN for functional classification has been demonstrated, for an offline motor-classification task. The implementation described in Section V could be incorporated to enable offline BCI applications such as choice-selection [6] and could potentially be adapted for other BCI tasks.

The results suggest that all three of the tested models are potentially viable for applications such as BCI requiring functional classification of fNIRS signals. All of the models achieved average classification accuracy of approximately 70%, which is conventionally considered the baseline requirement for a functional BCI system [31], and up to 84% for some subjects. It is possible that large variation in performance found between subjects was caused by the effect on fNIRS signal quality of hair colour and density, due to the high absorbance of dark hair to NIR light. It was noted that three of the four lowest performing subjects in this study were those with the most hair. By contrast, the results presented in [29] for a similar motor task, found an average F-score of 82% over eleven subjects, and far lower between-subject variation. The data in that study were collected with a NIRx Sport wearable fNIRS device with in-built ‘variable tension spring holders designed to improve source detector coupling’. This demonstrates the importance of specialised hardware for functional classification with fNIRS.

In the subject-independent training scheme, the *3D CNN + Block* model achieved a significantly higher F1 score than either of the other models. This finding supports previous research demonstrating the additional benefit of spatial feature extraction with CNNs [7], [28], [29]. However, no statistically significant difference was found between the F1 scores of the three models in the mixed subjects scheme i.e when data from the test subject also appeared in the training set. To understand why this might be the case, consider how features are computed in the 3D CNN: the same filters are applied to local groups of measurements at multiple positions in the input space, and therefore variation in the position of the functional haemodynamic response in relation to the array (caused by differences in both individual physiology and probe placement) has relatively small effect on the resulting feature space. In the case of the 1D CNN however, each chromophore time-series is taken as an independent variable, and therefore the features that are learned are specific for each position in the voxel space.

It is possible that the difference in the models' performances emerged only in the subject-independent scheme because in the mixed subjects scheme, the models could identify subject-specific markers in the data, which were also present in the test data, and were directly indicative of where to locate the relevant haemodynamic response. Although subjects were given the opportunity to take a break and remove the fNIRS cap between each run, most chose not to, and those who did took only one break, after the 3rd or 4th run. Therefore, for every subject there was a somewhat consistent relationship between the position of the HD probe and the position of the functional motor response, so if the network could determine which subject an example had come from, it could then rely on a single channel or small set of co-located channels to make the classification, which is easily learnable by the 1D CNN (i.e. without positionally invariant feature extraction). In the subject independent scheme however, the models were not trained on any examples from the test subject and therefore had no ‘markers’ by which to distinguish probe/FHR position, and had to rely purely on generalised features.

The considerably higher accuracy of all models in the mixed subjects scheme suggests that the models were relying partly on subject-specific features to improve classification, and this finding is in agreement with other subject-independent BCI studies such as [27] who found that their subject-independent classifier was outperformed by a simple LDA mixed subjects classifier with relatively few training examples. While the preprocessing pipeline in this study was designed to remove session- or subject- specific ‘markers’ from the data, such as the magnitude and variance of specific channels (corrected by z-score normalisation and interpolation of low quality channels) this is difficult to do completely, and the residual subject ‘markers’ clearly biased the classifiers in the mixed subjects training. It is for this reason that it is relevant to report in fNIRS BCI studies both the training paradigm (mixed subjects/subject independent) and also details of how the fNIRS probe was positioned, and whether it was repositioned or adjusted between experimental runs.

While previous work on subject-independent fNIRS BCI [27] has shown the efficacy of temporal CNNs, it has not reported the effect of spatial convolutions. On the other hand, previous work on spatial CNN for fNIRS BCI [7], [28], [29] has not highlighted the difference between mixed subject and subject-independent training. One contribution of this work therefore, is to indicate the specific benefit of positionally invariant feature extraction for session- and subject- independent BCI, and to propose an intuitive implementation for passing spatially arranged HD-fNIRS measurements to a spatial-temporal CNN. If the *3D CNN + Block* method indeed confers a greater benefit in functional classification when there is greater uncertainty in the positioning of the probe in relation to the haemodynamic response, then it could be a useful technique for developing generalised, subject- and session- independent classification models. One of the primary benefits of fNIRS for neuroimaging is that it can be applied in a naturalistic setting, and potentially, as recent work has highlighted, in a user-applied setting (e.g. in the home of a patient) [32]. In such cases there is likely to be considerable variation in probe placement between sessions, and data-driven models designed to account for this variation could improve the usability and accessibility of such systems.

The primary limitation in this study is the small number of subjects (n = 7). Intuitively, a more diverse training set would be expected to make it more difficult for the model fit to subjects, and make the differences in probe vs haemodynamic response position more continuous, and could therefore increase the benefit of positionally invariant feature extraction. Subsequent research in this subject may seek to prove or disprove this hypothesis, as well as to replicate these results over a larger and more diverse dataset. Additionally, conducting data collection with each subject over multiple sessions would help to separate the effects of subject-fitting (e.g. learning individual differences in haemodynamic strength, shape, position - potentially desirable) to session-fitting (e.g. learning arbitrary characteristics of a particular session i.e. source intensity, probe placement - not desirable).

This work highlights the crucial and often overlooked role that data structure can play in fNIRS processing and classification. As previously discussed, neural network models trained on HD-fNIRS data are increasingly prevalent in BCI research due to their high performance compared to traditional ML. In addition to classification, neural network-based models have been applied in multiple stages of fNIRS processing including removal of systemic physiological signals and motion artefact correction [33], creating the possibility of a modular end-to-end network pipeline for fNIRS processing and classification. The data structure is the link between probe design and network which can implicitly confer relational information (regarding channel position & SDS in this study) to the network in addition to the individual fNIRS signals. In this work a specific data arrangement afforded by the standard HD-array was identified, and showed how this choice afforded additional opportunities for data visualisation, augmentation and feature extraction. Another benefit of the Block technique is that it can be easily and intuitively expanded to additional data channels e.g. raw intensity and phase measurements from multiple fNIRS wavelengths or modulation frequencies, or hybrid fNIRS/EEG. Other specific SD configurations afford different techniques for processing data, each with their own costs and benefits. For example, Blaney et al. [34] have proposed the dual slope method, a specific arrangement of two pairs of sources and detectors to form four multi-distance channels which, taken together, can suppress instrumental artefacts and increase sensitivity to brain tissue. This and other techniques should be investigated and compared where possible. Given the diversity in configurations among state-of-the-art wearable high-density systems, it could also be beneficial to investigate a generalisable interpolation strategy which accounts for differences in SDS, and could therefore allow data from multiple systems to be used together for training a single model.

## IV. Conclusion

In this study, the utility of spatial-temporal-CNN for HD-fNIRS-BCI was investigated. Specifically, the performance of three combinations of fNIRS data structures and CNN architectures was compared in a three-class motor classification paradigm. The same input data and processing techniques were used in each case, so that the only factors in differences in performance were the structure of the data and the feature extraction technique employed in the first layer of the network. While previous studies have documented similar techniques, we believe that this is the first to present a direct comparison between 1D (temporal) & 3D (spatial & temporal) convolutions for functional classification. Applying 3D convolutions to the proposed *Block* representation of HD-fNIRS signals resulted in a statistically significant increase in the F1 score of a subject-independent BCI model, compared to both the 1D CNN, and another 3D CNN trained on a different data structure. These results suggest that the combination of spatial-temporal-CNNs with HD-fNIRS data affords a specific benefit for subject- and session- independent fNIRS BCI, and is worth further investigation. Furthermore, the significant difference in performance between the two 3D CNNs reveals that the effect was not due only to the model, but to the combination of the model with the data structure, highlighting the interdependence of probe design, data structure, and network architecture for data-driven fNIRS BCI.

## V. Materials and Methods

### A. Subjects

Seven human subjects (six male, one female, aged 36 $\pm$ 8 years) were recruited for the experiment. Subjects with little, light, thin, or no hair were preferentially selected, because fNIRS signal quality can be detrimentally affected by thick or dark hair, which is highly absorbent of NIRS light. Specialised hardware and probing techniques to overcome this problem are the subject of ongoing research [35], but are beyond the scope of this research. Ethical approval was obtained as part of the Programme of Work ERN_17-0544P at the University of Birmingham, U.K. (reference ERN_17-0544AP4), with all subjects providing written informed consent prior to participating.

### B. Experimental Design

Data were collected while subjects completed a finger-opposition exercise, in which there were three classes of activity: left-hand finger-opposition, right-hand finger-opposition, and rest. Each experimental run consisted of 16 task periods (8 left hand finger-opposition and 8 right hand finger-opposition) of 10 seconds, with a 15 s rest period after each. Additional 30 s rest periods were added at the start and end of each run.

Instructions to perform the finger-opposition task with either the left or right hand, or to rest, were given on a screen for feet in front of the subject, and additionally spoken aloud (“left”, “right”, “rest”) by the instructor. The order of the task blocks was pseudo-randomised (beginning with left) to avoid anticipatory effects and frequency correlation with systemic haemodynamic signals (respiratory and cardiac), which could artificially improve classification. Subjects each completed five runs of the experiment, each run taking approximately 10 minutes. The subjects were given the option to take a break and remove the fNIRS cap between each run.

The finger-opposition task required tapping the tip of the thumb against the tip of each finger in the following order: first, second, first, third, first, fourth, first, fifth, repeat. Before the experiment began, the instructor showed the subjects the finger-opposition pattern, and they were given thirty seconds to practise it. The pattern, which is more complex than a standard finger-tapping or hand-gripping action, was chosen to induce activation in the pre-motor cortex, which is involved in motor action planning, in addition to the primary motor cortex. During the experiment, subjects sat in a comfortable chair in a normally lit room with their eyes open, and their arms resting on the arms of the chair.

### C. Data Collection

The fNIRS data were collected using a dual-module Imagent 2 (ISS, USA) FD-DOS system, with thirty-two laser diode emitters (sources) ($\lambda 1 = \text{690}\,nm, \lambda 2 = \text{830}\,nm$, intensity modulated at 141 MHz) and thirty photo-multiplier tube (PMT) detectors, for a total of 440 measurement channels, sampled at 39 Hz. The sources and detectors were arranged in two HD grids, shown in fig, centred over the left and right primary motor cortices (10–20 positions C3 and C4). The sources and detectors were placed within two 3D-printed flexible mounts within a 5 mm thick neoprene swimming cap and secured in place with spring clamps figure.

### D. Preprocessing

The complex frequency-domain measurements were automatically converted into amplitude and phase components by the ISS system. Subsequent preprocessing was done using the open source neuroDOT Matlab package [36]. Channels with SNR (calculated as std/mean) of $>=7.5$ were marked as poor quality. Trials in which $>\!30\%$ of channels with SDS $< \text{30}\,mm$ were poor were rejected, because this is likely caused by errors in cap placement. A total of two runs were rejected.The data from each remaining run were converted to difference measurements with respect to the initial and final rest periods. Channels with SDS $>30\,mm$ were discarded, as the SNR was consistently poor across all subjects. It is not noted that the NN3 (39 mm) channels could be incorporated in the data structures described below, if detectors with greater dynamic range were used.

Optical Density and phase shift were calculated as:

\begin{align*}
\Delta OD = -log\left(\frac{i}{\mu _{i}}\right) \tag{1}
\\
\Delta ph = ph - \mu _{ph} \tag{2}
\end{align*}

where $i$ and $ph$ are the measured intensity and phase data, and $\mu$ is the mean of the time-series across the entire run. A bandpass filter at 0.01–0.5 Hz was applied to remove confounding physiological perturbations. Measurements were converted to HbO and Hb concentrations by generating a semi–infinite model with NIRFAST and inverting the jacobian (sensitivity matrix), however it was noted that comparable classification results were achieved by passing the processed intensity and phase measurements directly to the classifier. Data were resampled at 2 Hz, and the data from each channel were standardised by taking the z-score:

\begin{equation*}
Z = \frac{x - \mu }{\sigma } \tag{3}
\end{equation*}

where $x$ is the measured value, $\mu$ and $\sigma$ are the mean and standard deviation of the time-series across the entire run. Channels marked as poor quality were replaced by an average of up to eight surrounding good channels of the same chromophore and NN (those immediately adjoining and diagonal in the *Block* representation). The processed fNIRS data was split into windows of twenty frames (ten seconds) per example, labelled according to the instruction on the screen at the time (left-hand finger-opposition, right-hand finger-opposition, or rest). The final dataset consisted of 1023 labelled examples from thirty-three runs (five for each of seven subjects, minus two rejected).

### E. Data Structures

#### 1) Block

The proposed model uses a *Block* arrangement, which is designed to exploit the repeating geometry of the HD-array to improve the efficiency of spatial feature extraction using convolutional filters. Firstly, the HbO & Hb measurements were grouped by SDS, into nearest neighbour 1 (NN1, 13 mm SDS) and nearest neighbour 2 (NN2, 29 mm SDS). The NN2 measurements were further divided into horizontally and vertically oriented channels. Within these groups, data from each time-step were arranged in grids according to their position on the probe, identified as the central point between the source and detector (shown as yellow squares in Fig. 6). The six grids were then stacked along the channel dimension, as shown in Fig. 8, similarly to the color channels in an image. Because the HD-array creates a grid of voxels, the fNIRS measurements can be represented as images without interpolation, producing a set of topographic images of haemodynamic changes. with consistent corresponding voxels, and different depth sensitivity profiles, determined by SDS. As noted above, only NN1 and NN2 were used in this study, due to the limited dynamic range of the PMTs, however this technique could straightforwardly be extended to include the NN3 measurements.

**Figure 7.**
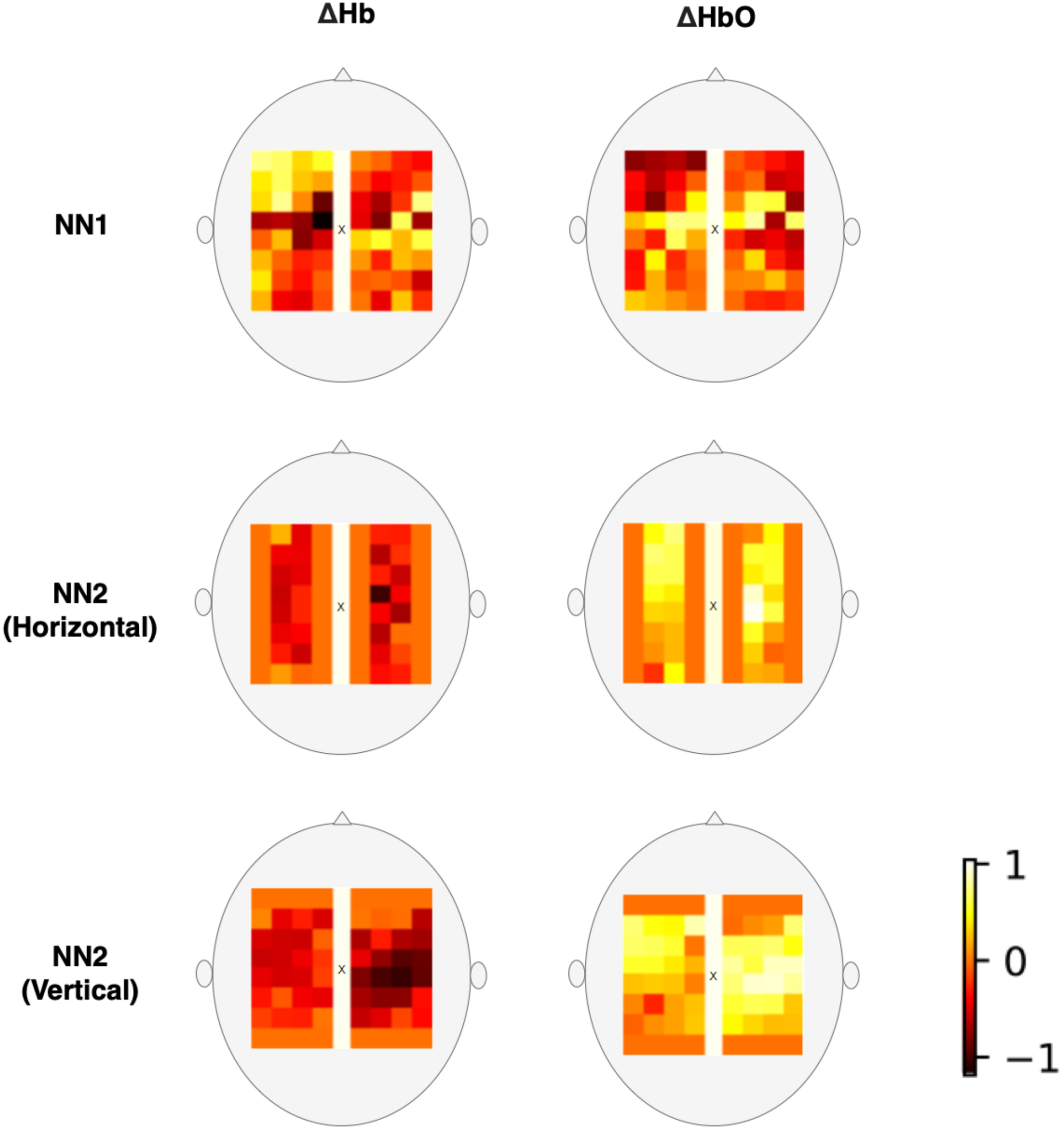
Spatially organised high density Hb & HbO measurements, block-averaged over a left-handed finger tapping task period, arranged according to channel position.

**Figure 8.**
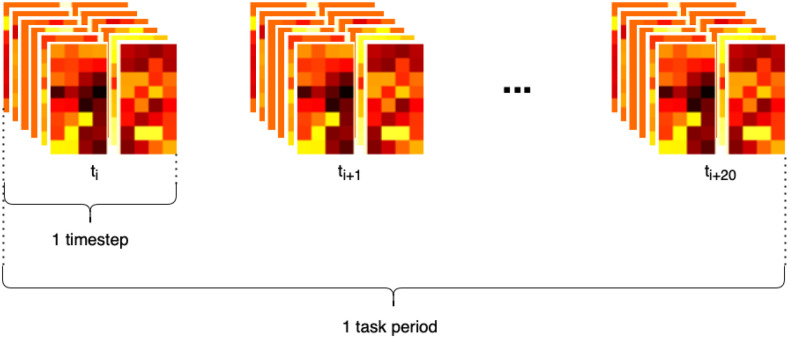
High-density HbO & Hb measurements in the *Block* structure used to train a 3D CNN.

As the task-averaged measurements in Fig. 7 illustrate, this arrangement captures spatial information relating to functional activation. An increase in HbO and decrease in Hb in the NN2 measurements can be observed, with their greatest changes located over the C4 and C3 regions of the motor cortex, respectively. This response is typical of the haemodynamic response to a left handed motor action (finger-opposition in this case).

#### 2) Flat

**Figure 9.**
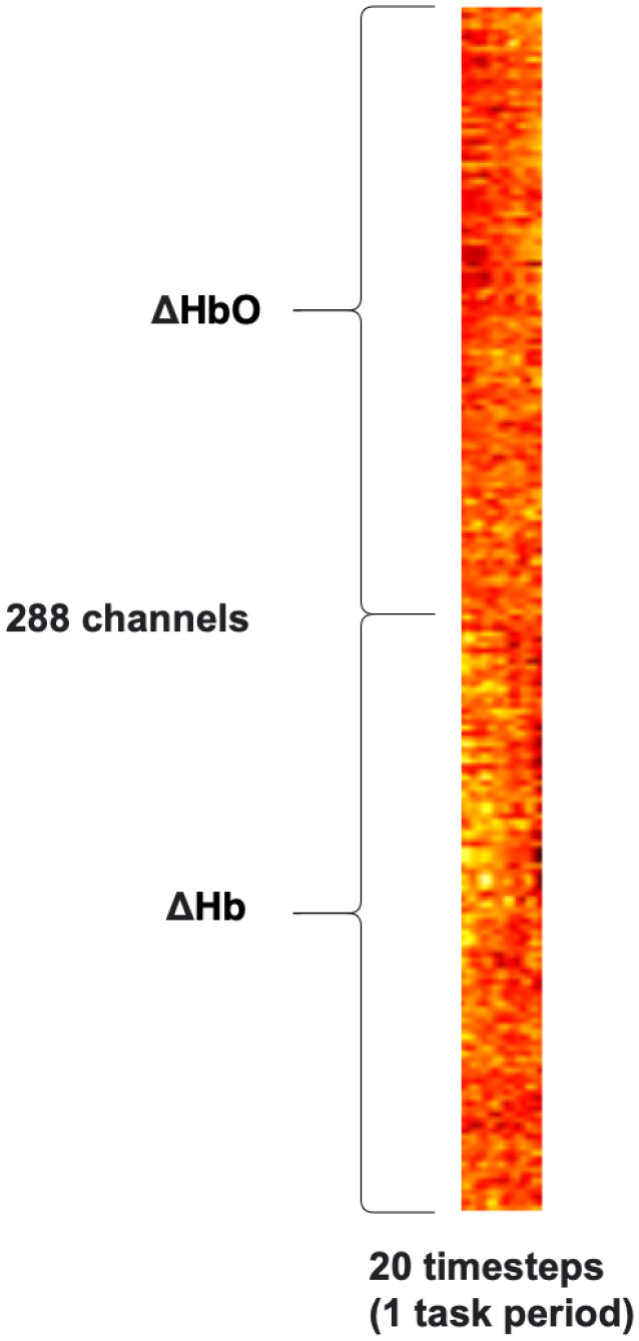
High-density HbO & Hb Measurements in the *Flat* structure used to train a 1D CNN.

fNIRS data from each task/rest period is represented as an (n x m) matrix where n is the number of SD pairs x2 (Hb & HbO), and m is the number of time-steps (20 in this case: 10 seconds x 2 Hz), as shown in Fig. 9. This representation was used to train a 1D CNN which applied convolutional filters across the time dimension only, treating each input measurement as an independent variable. This model is included for comparison as it is the standard approach to training CNNs with fNIRS data, and is demonstrated in [23], [24], [26], [37].

#### 3) S-By-D

**Figure 10.**
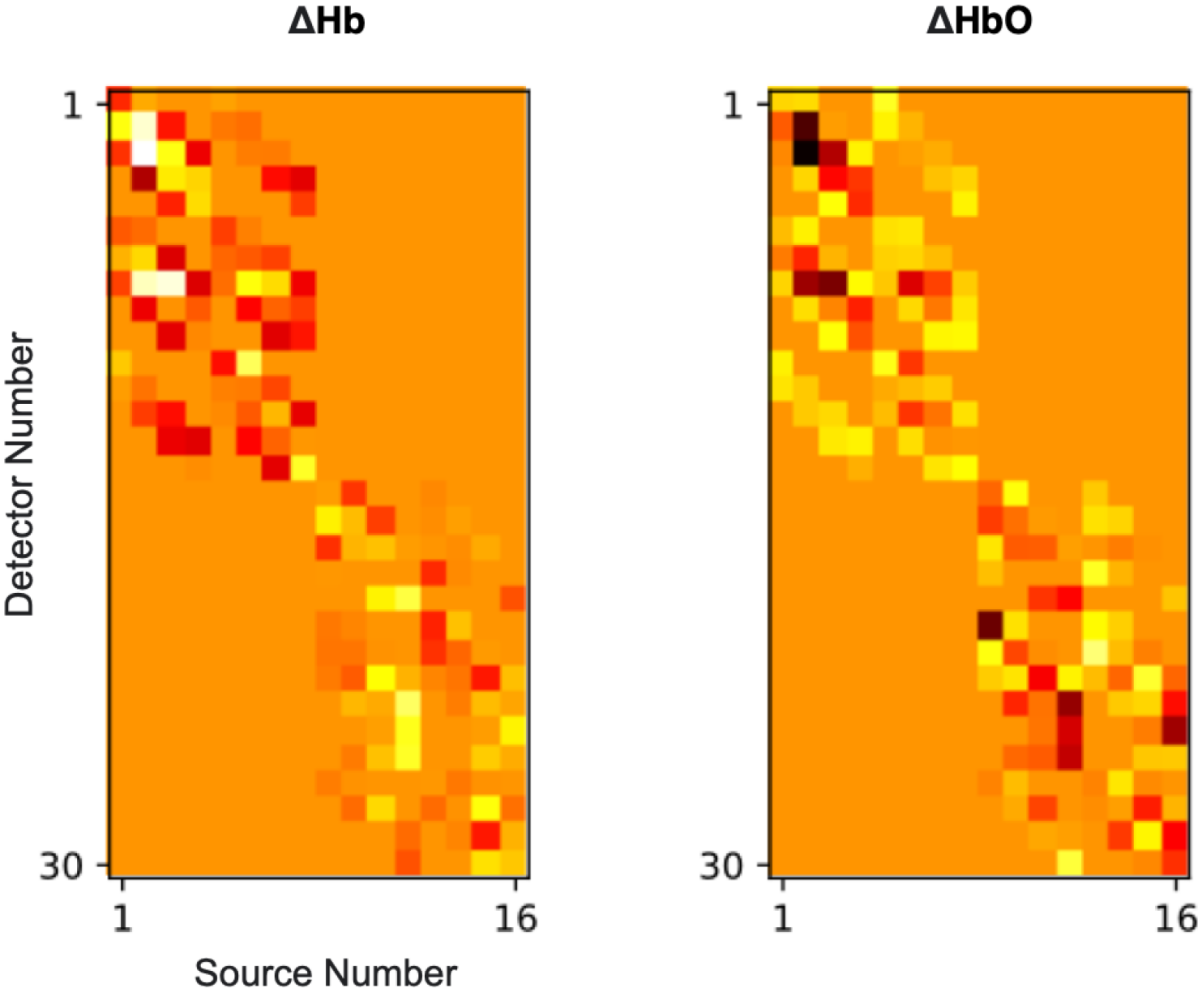
High-density Hb & HbO measurements, block-averaged over a left-handed finger tapping task period, arranged according to source and detector number.

**Figure 11.**
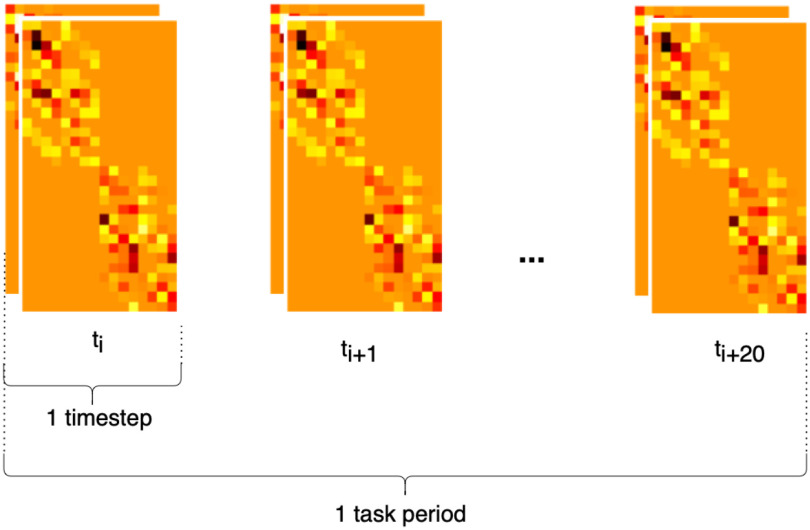
High-density HbO & Hb measurements in the *S-by-D* structure used to train a 3D CNN.

The *S-by-D* arrangement is a straightforward, non-probe-dependent way of presenting fNIRS measurements as an image. Hb & HbO measurements from each time-step were arranged in (s x d) matrices, where s is the number of sources and d is the number of detectors, as shown in Fig. 10, and concatenated along channel and temporal dimensions, as shown in Fig. 11, This representation is used just like *Block* to train a 3D convolutional network. The *S-by-D* representation is not expected to confer a specific benefit for spatial feature extraction, rather it is included in for comparison to separate the effects of the architecture alone (3D vs 1D CNN) from the effect of the architecture + data structure combination.

## VI. Data Augmentation

A novel data augmentation technique was implemented to increase the number of training examples in the *Block* training set, enabled by the spatial arrangement. Each example labelled as either right- or left- finger-opposition was flipped along the vertical axis, so that the left motor cortex measurements were positioned on the right hand side and vice versa. The flipped examples were labelled as the opposite task state. This technique is based on the assumption that the contralateral motor cortex activation is generally dominant, and therefore a reversed image of left-hand tapping activation is a sufficiently realistic simulation of right-hand tapping activation to support the training of the classifier. As the data were already in a spatially resolved format, the number of training examples could be doubled in a single line of python code.

## VII. Model Architectures

CNNs generally consist of a series of convolutional layers for feature extraction, followed by fully connected layers for classification. A fully connected layer is the most basic neural network component. It consists of a series of nodes, each of which computes a weighted sum of all the input values plus a bias term, before applying a non-linear activation function. The weights and biases of the nodes are randomised at the beginning of the training process, and then adjusted to minimise a loss function calculated between network output probabilities and the ground truth label of each example. A convolutional layer similarly uses trainable weights and biases to compute node values, however the weights are grouped into filters, which are cross-correlated with the input data to produce a set of n spatial feature maps, where n is the number of filters applied. Where the input data has multiple channels, a separate set of filter kernels is trained for each channel. A key feature of the convolutional operation is that it enforces *positional invariance:* the same set of features (transformations) are calculated at each position in the input space, and the derivatives calculated during back-propagation are used to update the same filters regardless of where the feature was detected, making it very efficient to learn spatially recurring features. Thus, while any transformation performed by a convolutional layer is possible to perform with a FC layer, the FC layer, which learns the relationships between each set of input values independently, requires a far greater number of parameters and far greater number of training examples to learn. This is why CNNs generally outperform even very large FC networks for image, video, and time-series classification.

The proposed model combines 3D CNN architecture with the *Block* data structure described in Section V-E. The proposed CNN consists of an initial 3D convolutional layer for spatial and temporal feature extraction, followed by two fully connected layers for classification. The number of each type of layer (convolutional and fully-connected), and the size of the layers, in addition to other hyper-parameters, were determined by grid search (see Section VIII), and the optimal numbers may be different depending on the size of the dataset (larger datasets generally facilitate training larger networks).

The *Block* structure is designed to allow the 3D CNN to efficiently learn features in the fNIRS data relating to the functional haemodynamic response, which may occur at multiple locations across the array. Furthermore, because the different NN measurements a separated between channels, the convolutional filters of each channel are allowed to specialise to represent the different relationships between the SD positions and the regions of sensitivity for each SDS. For e.g. the network could learn to ignore residual systematic changes which are present simultaneously in both NN1 and NN2 channels.

For comparison, two other models were trained on the same dataset: a 1D CNN trained on *Flat* data structure, and another 3D CNN with the same architecture as the proposed model, but trained on *S-by-D* data structure.

## VIII. Hyper-Paramenter Tuning

**Figure 12.**
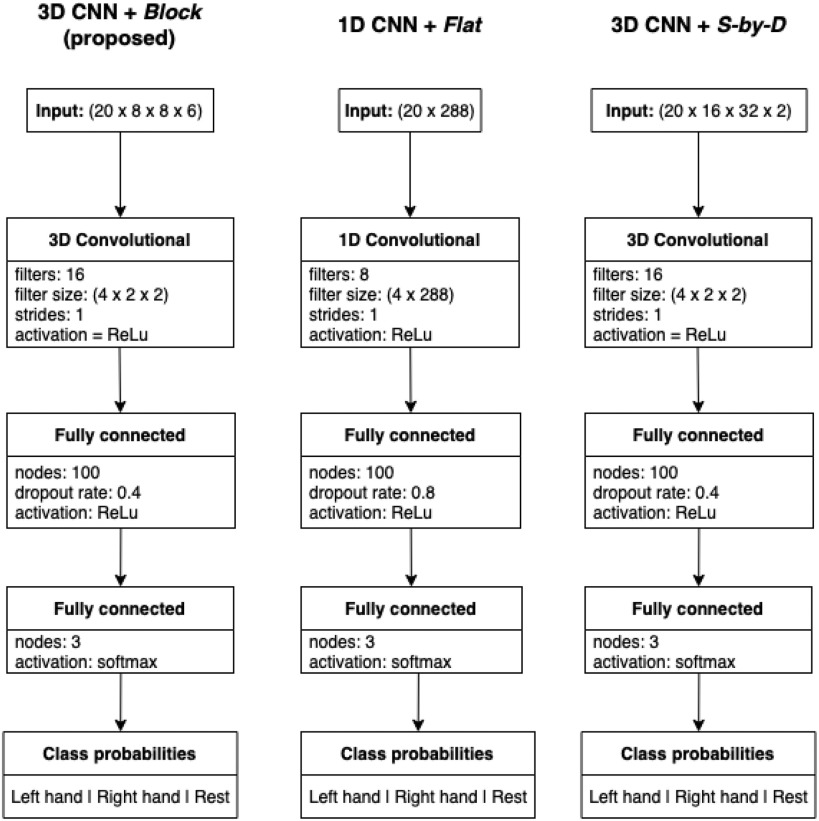
System diagrams of the three models compared for functional classification.

The hyper-parameters of each model were determined Empiricall using a class-stratified, group-based five-fold cross-validation grid-search, meaning that for each combination of hyper-parameters, the training examples were split into train and validation sets such that no collection session's data appeared in both sets, and so that the class distribution was preserved. The test data session from each subject that the models were evaluated with was held out of this process. The tested values for each parameter are listed in Table 3, with the selected parameters shown in **Bold**. The system diagrams for each model are shown in Fig. 12.

**TABLE 1:** Classification Results for Each Subject Using the Mixed Subjects Training Scheme

	**Sub 1**	**Sub 2**	**Sub 3**	**Sub 4**	**Sub 5**	**Sub 6**	**Sub 7**	**Avg.**
**3D CNN + Block**	**70.9$\pm$5**	**45.6$\pm$4**	**82.3$\pm$3**	**84.4$\pm$3**	**78.6$\pm$4**	**56.5$\pm$3**	**66.2$\pm$4**	**69.2$\pm$4**
**3D CNN + S-by-D**	**74.4$\pm$5**	**44$\pm$4**	**81.6$\pm$2**	**84.3$\pm$3**	**82.3$\pm$5**	**54.8$\pm$4**	**68.7$\pm$3**	**70$\pm$4**
**1D CNN + Flat**	**75$\pm$4**	**44$\pm$4**	**83.6$\pm$4**	**85.2$\pm$4**	**81.4$\pm$5**	**61.4$\pm$5**	**62.6$\pm$6**	**70.5$\pm$5**

**TABLE 2:** Classification Results for Each Subject Using the Subject-Independent Training Scheme

	**Sub 1**	**Sub 2**	**Sub 3**	**Sub 4**	**Sub 5**	**Sub 6**	**Sub 7**	**Avg.**
**3D CNN + Block**	**53.7$\pm$2**	**50.8$\pm$3**	**69.6$\pm$4**	**72.9$\pm$2**	**69.9$\pm$3**	**40.6$\pm$2**	**50.7$\pm$3**	**58.3$\pm$3**
**3D CNN + S-by-D**	**46.5$\pm$2**	**45.1$\pm$2**	**68$\pm$5**	**71.1$\pm$3**	**69.2$\pm$3**	**39.4$\pm$3**	**50.2$\pm$2**	**55.6$\pm$3**
**1D CNN + Flat**	**47.3$\pm$3**	**47.9$\pm$5**	**68.9$\pm$5**	**67.7$\pm$5**	**71.2$\pm$7**	**39.4$\pm$4**	**47.9$\pm$4**	**55.7$\pm$5**

**TABLE 3:** The Hyper-Parameter Values Tested for Each Classification Model. Selected Values Are Shown in **Bold**

	**Block + 3D CNN**	**Flat + 1D CNN**	**S-by-D + 3D CNN**
**n conv layers**	**1**, 2, 3	**1**, 2, 3	**1**, 2, 3
**n filters**	4, 8, **16**, 32	4, **8**, 16, 32	4, 8, **16**, 32
**filter temporal size**	2, **4**, 10	2, **4**, 10	2, **4**, 10
**n FC layers**	1, **2**, 3	1, **2**, 3	1, **2**, 3
**FC size**	25, **100**, 200	25, **100**, 200	25, **100**, 200
**Dropout rate**	0.2, **0.4**, 0.8	0.2, 0.4, **0.8**	0.2, **0.4**, 0.8
**Batch size**	25, **50**, 100	25, **50**, 100	25, **50**, 100
**n epochs**	30, **60**, 100	30, **60**, 100	30, **60**, 100
**Learning rate**	0.1, **0.01**, 0.001	0.1, **0.01**, 0.001	0.1, **0.01**, 0.001

The optimised models shared the same values for many hyperparameters including batch size, training rate, training epochs, and the size of the convolutional kernel on the time dimension. This makes sense because the validation data was equivalent in terms of magnitude, distribution, and temporal resolution.

## IX. Training

Two different training schemes were tested: *mixed subjects* and *subject-independent*. In the mixed subjects scheme, models were trained on examples from all subjects and tested on examples from a held out collection session. In the subject independent scheme, models were trained on examples from six of the seven subjects, and tested on examples from the held out subject. The between subjects scheme is designed to emulate subject independent BCI, in which a pre-trained BCI system can function with a new user without an initial data collection/calibration session.

## X. Classification Analysis

F1 score, averaged across the three classes (left hand, right hand, rest), was selected as the primary assessment metric, and used in all statistical analysis, as it balances type-1 and type-2 errors, a crucial consideration in classification tasks where the class distribution is unequal. To determine the effect of the different models on classification performance, we perform statistical tests where the null hypothesis is that the choice of model has no effect on the distribution of results. Normality was tested using Shapiro Wilk test. Because there were two independent variables in the experiment (subject and classification model), a two-way analysis of variance (ANOVA) test was used to compare the F1 scores of the models, while controlling for variation between subjects.
